# Morphological dataset of aboveground macrofungal communities within different forest conversion stages in the Eifel National Park in Germany

**DOI:** 10.1016/j.dib.2018.10.094

**Published:** 2018-10-26

**Authors:** Peggy Heine, Jonas Hausen, Richard Ottermanns, Andreas Schäffer, Martina Roß-Nickoll

**Affiliations:** Institute for Environmental Research, ABBt Aachen Biology and Biotechnology, RWTH Aachen University, Worringer Weg 1, 52074 Aachen, Germany

## Abstract

This dataset includes 235 aboveground macrofungal species observed at 15 sampling sites, which are associated with five different forest conversion stages. We used a space-for-time substitution approach to represent a forest conversion from Norway spruce (*Picea abies*) to European beech (*Fagus sylvatica*) through three different, widely used management practices. In addition to the results of 75 macrofungal field surveys, this data article includes information about site characteristics, vegetation structure, and observation frequencies. A multivariate statistic and myco-ecological interpretation of the macrofungal dataset is presented in an associated research article entitled “Forest conversion from Norway spruce to European beech increases species richness and functional structure of aboveground macrofungal communities” (Heine et al., 2019) [1].

**Specifications table**

**Table:** 

**Subject area**	Biology, Forest Management
**More specific subject area**	Ecology, Mycology
**Type of data**	Excel file with four tables
**How data was acquired**	Aboveground myco-ecological surveys, microscopic and macroscopic determination, lab work
**Data format**	Raw (species data) and analyzed (environmental data)
**Experimental factors**	All visible sporocarps were identified at sampling site level
**Experimental features**	Total observed species richness, community composition, functional group species richness, environmental variables, vegetation abundance data
**Data source location**	Germany (GPS coordinates in associated excel file)
**Data accessibility**	Dataset is provided with this article
**Related research article**	“Forest conversion from Norway spruce to European beech increases species richness and functional structure of aboveground macrofungal communities“ [1]

**Value of the data**

• This dataset includes 230 aboveground macrofungi at species-level and 5 species at genus-level together with their functional group. This information can be used to study ecological functioning of different macrofungal communities.
• The species list will be suitable to enhance the understanding and interpretation of forest conversion management practices.
• Due to the stratified sampling design, the dataset is useful for comparison with other forest management studies.
• The additional site characteristics can help to interpret effects of environmental variables on macrofungal communities and can be utilized to build forest models.

## 1 Data

All observed data are available as Supplementary material in this data article. The name of the excel file is “mmc2.xlsx” and it consists of four excel tables (“macrofungi”, “environmental variables”, “vegetation”, and “observation frequencies”):

i. The first excel table “macrofungi” includes all macrofungal species observed in 15 sampling sites. The sheet contains the functional group [ectomycorrhizal fungi (EMF), pathogenic fungi (P), wood-decaying fungi (WDF), litter-decaying fungi (LDF), wood- or litter-decaying fungi (WDF/LDF), fruit-decaying fungi (FDF), wood-decaying fungi/pathogenic fungi (WDF/P), and dung-decaying fungi (DDF)] of each fungal species (Column A), the phylum [Ascomycetes (A), Basidiomycetes (B)] (Column B), the Latin species name with author (Column C), the abbreviation of the species name (Column D), the threatened status according to the red list of the federal state North Rhine-Westphalia from 2011 [2] (Column E), the threatened status according to the red list of Germany from 2016 [3] (Column F), the order of the fungal species (Column G), family of the fungal species (Column H) and all presence-absence data [presence (+) and absence (.)] within all sampling sites; norway spruce (*sp1-3*), salvage-logged windthrow (*wtminus1-3*), unmanaged windthrow (*wtplus1-3*), close-to-nature managed spruce/beech mix (*spb1-3*), and European beech (*b1-3*) (Column I–W).
ii. The second excel table “environmental variables” holds sampling sites (Column A), forest conversion stage (Column B), abbreviation of stage (Column C), forest district (Column D), management history (Column E), dominant tree species (Column F), elevation in meter above sea level (m a.s.l.) (Column G), latitude (Column H), longitude (Column I), pH topsoil (Column J), pH litter (Column K), C/N topsoil (Column L), C/N litter (Column M), %total carbon topsoil (Column N), %total carbon litter (Column O), %total nitrogen topsoil (Column P), %total nitrogen litter (Column Q), Ellenberg indicator value (EIV) for light availability (Column R), EIV for soil reaction (Column S), EIV for nutrient availability (Column T), EIV for temperature (Column U), EIV for soil moisture (Column V), average surface temperature difference (∆T) of the year 2012 (Column W), average surface humidity difference (∆H) of the year 2012 (Column X), fungal species richness (Column Y), plant species of light-demanding species (Column Z), plant species richness (Column AA), F:P (Fungal:Plant) ratio (Column AB), canopy closure (Column AC), species richness of basidiomycetes (Column AD), species richness of ascomycetes (Column AE), species richness of wood-decaying fungi (Column AF), species richness of litter-decaying fungi (Column AG), species richness of ectomycorrhizal fungi (Column AH), species richness of pathogenic fungi (Column AI), species richness of dung-decaying fungi (Column AJ), species richness of fruit-decaying fungi (Column AK), species richness of wood-decaying fungi/ pathogenic fungi (Column AL), and species richness of wood-decaying fungi/litter-decaying fungi (Column AM).
iii. The third excel table “vegetation” contains abundance values of all observed vascular plants within the 15 sampling sites according to the Braun–Blanquet scale [4] modified by Reichelt and Wilmanns [5]. The table is ordered in vegetation layer [Herb (H), Shrub (S), Tree (T)] (Column A), Latin name (Column B) and vascular plant abundance value according to r, +, 1, 2, 3, 4, and 5 within all sampling sites; Norway spruce (*sp1-3*), salvage-logged windthrow (*wtminus1-3*), unmanaged windthrow-(*wtplus1-3*), close-to-nature managed spruce/beech forest mix (*spb1-3*), and European beech - (*b1-3*) (Column C–Q).
iv. The fourth excel table “observation frequencies” summarizes all survey data of the years 2010–2012 and shows the number of replicate sites, which were surveyed at the specific dates. Every sampling site was visited 5 times in the entire observation period of 3 years.

## 2 Experimental design, materials, and methods

### 2.1 Experimental design

The topsoil sampling was conducted at fourteen sampling sites in the Eifel National Park (50°34′12.60″N 6°21′38.50″E) and at one sampling site in the neighbouring community forestry Monschauer Stadtwald (50°31′51.43″N 6°18′53.93″E) in Germany. We used a space-for-time substitution approach [6] to obtain five different temporal stages as representatives of the forest conversion from Norway spruce (*Picea abies*) to European beech (*Fagus sylvatica*) across three forest management practices: Salvage-logged windthrow, unmanaged windthrow, and close-to-nature management with subsequently beech tree underplanting. For the initial and final vegetation types of the spruce forest conversion process, we used even-aged, ~70-year-old Norway spruce forests, which were managed with silvicultural strategies until 2004, and uneven-aged, >120-year-old-growth European beech forests that are likely to have been formed naturally in the last 100 years.

**Fig. 1:**
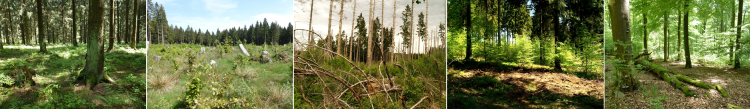
Impressions of the five forest conversion stages (from left to right): Norway spruce forest, salvage-logged spruce windthrow, unmanaged spruce windthrow, close-to-nature managed spruce/beech mixed forest, and European beech forest.

Overall, a stratified sampling design was applied on three spatially independent replicate sampling sites (10 m × 10 m) for every forest conversion stage. In total, five different forest conversion stages were *a priori* classified through information about their vegetation composition, site history and management history to guarantee the following homogeneous habitat structures (Fig. 1):

(I) Norway spruce sites (*sp1-sp3*): Single-species, even-aged, ~70-year-old Norway spruce forests, used for commercial wood production until 2004.
(II) Unmanaged windthrow sites (*wtplus1-wtplus3*): Single species Norway spruce forests that experienced high levels of windthrow by windstorm Kyrill in 2007. Thrown spruce trees were left behind at the time of the observations, CWD (≥ 7 cm) had mostly decay class II (bark and twigs present, solid).
(III) Salvage-logged windthrow sites (*wtminus1-wtminus3*): Single species Norway spruce forest, influenced by large-scale disturbance of the windstorms Wiebke (1990), Vivian (1990), and Kyrill (2007), followed by salvage logging due to the risk of bark beetle attacks. Thereby, all stems were removed, while both uprooted and rooted cut stumps were left.
(IV) Spruce/beech mixed sites (*spb1-spb3*): ~70-year-old Norway spruce forest, close-to-nature managed by selective cutting of single spruce trees and underplanting of European beech trees in 2007. The sampling site *spb1* was in the neighbouring community forestry Monschauer Stadtwald due to limited areas of similar historical and management conditions within the National Park area.
(V) European beech sites (*b1-b3*): Uneven-aged (multi-aged), old-growth beech forests with European beech as dominant tree species and without any management in a long period (>100 years). The sampling site *b1* was the oldest habitat with 191 years and characterized as a forest nature reserve (nature forest area) of North Rhine-Westphalia [7]. The other sampling sites *b2* and *b3* were ~120 years old.

## 3 Materials and methods

### 3.1 Site characteristics

All five forest conversion stages were represented by three replicated sampling sites. All 15 sampling sites (each 10 m × 10 m) were buffered by an appropriate distance (≥ 100 m) from each other. At five systematical sample locations in each sampling site (middle and each corner), topsoil (0–10 cm depth, soil core ø 5 cm) and aboveground litter (layer OL) were separately collected. The other part was crushed using a mortar and a pestle. The powder was dried at 105 °C (topsoil) or 80 °C (litter) for 48 h to measure the total carbon (C) and nitrogen (N) contents using dry combustion on an Elementar VarioEL v.4.01 (Hanau, Germany). All determinations were performed in duplicate.

In July/August of 2010 and 2011, cover values of vascular plant abundance were recorded in each sampling site using the Braun–Blanquet scale [4] in a modified version of [5]. Species taxonomy was documented according to 8 (2011). The vegetation data were mainly recorded to pre-classify the replicated sampling sites to represent the five forest conversion stages and to calculate Ellenberg indicator values (EIV). Average EIVs for light availability (EIVL), for soil moisture (EIVM), for temperature (EIVT), for soil reaction (EIVR), and for nutrient/soil fertility (EIVN) were computed for each sampling site [8]. In addition, the plant richness of light-demanding species (PRL) was calculated the mean number of plants observed in three replicates per stage indicating EIVL values from 7 to 9. We recorded the surface temperature [°C] of each sampling site on ground level with one data logger (OM-EL-USB-2, 2004-12, Omega Engineering Inc.). We used hourly recordings from 28th May to 16th September in the years 2011 and 2012. Subsequently, we calculated the difference of the daily surface temperature, defined as Δ*Ts* = *Ts* (max) − *Ts* (min), where *Ts* (max) and *Ts* (min) are the daily maximum and minimum surface temperature of each sampling site. Due to loss or damage of some data loggers in sites *spb1*, *spb3*, *b2*, and *wtminus1*, the data from both years were merged. Averages of the daily surface temperature of the recording time were used in the analyses. The same data mining was performed for the relative humidity [%] at ground level (∆Hs). Canopy closure was assessed by visual estimation at one point during the vegetation surveys in comparable weather conditions and expressed as the percentage of each sampling site. GPS coordinates and elevation [m a.s.l.] of each site were determined with a GPS navigator (Garmin eTrex Legend® HCx). An overview about the sampling locations can be found in the related research article [1].

### 3.2 Macrofungal sampling

We observed the presence of all epigeous macrofungi (Basidiomycetes and Ascomycetes), visible to the naked-eye [9], [10]. We examined sporocarps over 3 years (2010–2012) within the same sampling sites as the topsoil and litter samples were collected. Both windthrow sites were monitored for 2 years (2011–2012). Within each survey period, all sampling sites were sampled for ~3 h. Most of these sporocarps were photographed in their natural habitat and identified in situ, difficult species were collected to confirm their micro-morphological characteristics in the lab. Species identifications were performed using mainly [11], [12], [13], [14], [15], [16], [17], [18], [19], [20], [21]. Critical taxa were confirmed by experienced mycologists (see acknowledgments). The current nomenclature was validated using the Mycobank Database (www.mycobank.org; last accessed 26 March 2018). Myxomycetes and species observed outside the 10 m × 10 m sampling site were excluded, whereas taxonomically critical species and anamorph forms were included. Doubtful records are indicated by the abbreviation ‘cf.’ or ‘aff.’ in the species names. Fungi identified to the genus only are indicated ‘sp’. The threat status of each species was selected by red lists for fungi available for North Rhine-Westphalia [2] and for Germany [3]. We estimated the mean species richness by averaging the total number of fungi of the three replicated sites per each stage. We distinguished the fungal species based on similar functional roles in ecosystem processes [22] according to field conditions and literature [23], [24], [25] into eight different functional groups: wood-decaying fungi living on dead wood of branches, stumps, sticks, and trunks (WDF), litter-decaying fungi living on litter, needles on the ground (LDF), ectomycorrhizal fungi (EMF), pathogenic fungi (P), wood- or litter-decaying fungi (WDF/LDF), fruit-decaying fungi living on spruce cones and beechnuts (FDF), wood-decaying fungi existing on dead wood or living wood (WDF/P), and dung-decaying fungi (DDF). Any fungi with bryophyte- or pyrenomycete-macrofungus relationships were classified as EMF, such as *Rickenella fibula* or *Tremella globispora*.
