# Supplementary material for: Morphological dataset of aboveground macrofungal communities within different forest conversion stages in the Eifel National Park in Germany
Source: Data Brief. 2018 Oct 26;21:1151–6. doi: 10.1016/j.dib.2018.10.094 (PMC6231034; doi:10.1016/j.dib.2018.10.094)
Supplement: Supplementary file 1 — Supplementary material [file mmc1.docx]

**Conflict of Interest**

There are no conflicts of interest to publish this research paper.
